# Complete Genome Sequences of Ralstonia solanacearum Strains Isolated from Zingiberaceae Plants in Japan

**DOI:** 10.1128/MRA.01303-20

**Published:** 2021-01-28

**Authors:** Kazuhiro Iiyama, Sawa Kodama, Honoka Kusakabe, Yoriko Sakai, Mitsuo Horita, Kazutaka Yano, Htet Wai Wai Kyaw, Kenichi Tsuchiya, Naruto Furuya

**Affiliations:** a Laboratory of Plant Pathology, Faculty of Agriculture, Graduate School, Kyushu University, Fukuoka, Japan; b Institute for Agro-Environmental Sciences, National Agriculture and Food Research Organization (NARO), Ibaraki, Japan; c Kochi Agricultural Research Center, Kochi, Japan; University of Arizona

## Abstract

Here, we report the complete genome sequences of three Ralstonia solanacearum strains isolated from Zingiberaceae plants in Japan. The total genome sizes of these strains ranged from 5.87 to 6.05 Mb. Strains MAFF 211472, MAFF 211479, and MAFF 311693 each carried one chromosome and one megaplasmid. MAFF 311693 contained an additional 71.9-kb plasmid.

## ANNOUNCEMENT

Ralstonia solanacearum is a soilborne plant-pathogenic bacterium. In Japan, R. solanacearum strains infecting Curcuma alismatifolia (Zingiberaceae) were first reported in 1995 (1). Thereafter, the bacterium was isolated from other Zingiberaceae plants, including ginger (Zingiber officinale), myoga (Z. mioga), turmeric (C. longa), wild turmeric (C. aromatica), and zedoary (C. zedoaria) (2–4). Repetitive sequence-based PCR (rep-PCR) assays identified invasive strains from foreign countries (5).

Since not all R. solanacearum strains are pathogenic to Zingiberaceae plants (6), the genomes of bacterial isolates from Zingiberaceae plants were sequenced to determine their pathological potential. The R. solanacearum strains (phylotype I, race 4, biovar 4) used in this study were MAFF 211472, MAFF 211479, and MAFF 311693. MAFF 211472 was isolated from ginger in Nakamura, Kochi Prefecture, Japan, in 1997. MAFF 211479 was isolated from ginger in Kahoku, Kochi Prefecture, Japan, in 1997. MAFF 311693 was isolated from wild turmeric in Nago, Okinawa Prefecture, Japan, in 2016. The strains were preserved as freeze-dried cultures at Genebank in the National Agriculture and Food Research Organization (NARO, Tsukuba, Ibaraki, Japan). The freeze-dried cultures were rehydrated in the Laboratory of Plant Pathology (Kyushu University). The cultures were stored as freeze-dried cells and 20% glycerol suspensions at −70°C for long-term preservation and routine work, respectively.

The glycerol suspension was streaked onto Casamino Acid-peptone-glucose (CPG) medium (7), and the single colony isolation was repeated with CPG medium two to three times for genome extraction. Single colonies of R. solanacearum strains were cultured in CPG broth overnight at 30°C. Genomic DNA was extracted using the cetyltrimethylammonium bromide protocol (8). The extracted DNA was used on long-read and short-read sequencing platforms to generate hybrid assemblies using single-molecule real-time (SMRT) (PacBio, CA, USA) and Illumina (San Diego, CA, USA) sequencing, respectively. Library preparation and sequencing were performed by Novogene Co., Ltd. (Beijing, China).

For PacBio sequencing, SMRTbell libraries were generated using sheared template DNA, and SMRT sequencing was carried out on a PacBio Sequel platform, creating 178,820 (MAFF 211472), 137,360 (MAFF 211479), and 187,585 (MAFF 311693) subreads with average lengths of 9,231 bp, 9,513 bp, and 9,912 bp, respectively; the *N*_50_ values were 10,416 bp, 10,783 bp, and 11,237 bp, respectively.

For Illumina sequencing, we generated paired-end libraries (fragment size, ∼350 bp) using the NEBNext Ultra DNA library prep kit. Libraries were sequenced on an Illumina HiSeq 4000 sequencing system. After filtering with Trimmomatic v.0.32 (9), 5.24, 4.71, and 4.95 million 150-bp paired-end reads were used to generate hybrid assemblies for MAFF 211472, MAFF 211479, and MAFF 311693, respectively. Hybrid *de novo* assemblies were generated using Unicycler v.0.4.7 (10) with PacBio long reads and Illumina short reads. Default parameters were used for all software. The assemblies of MAFF 211472 and MAFF 211479 had total lengths of 6,053,933 bp with 66.7% GC content and 5,905,604 bp with 66.8% GC content, respectively. Both comprised two circular contigs, a chromosome and a megaplasmid. In MAFF 311693, the genome size was 5,869,920 bp with 66.9% GC content, and three circular contigs were generated, indicating that this strain carries an additional 71,852-bp plasmid.

The assemblies were annotated using DFAST (11), and the results are summarized in Table 1.

**TABLE 1:** Accession numbers, assembly metrics, and annotated features of the sequenced Ralstonia solanacearum strains

Strain^*a*^	GenBank accession no.	Genome assembly size (bp)	GC content (%)	No. of:						
				CDS^*b*^	16S rRNAs	23S rRNAs	5S rRNAs	tRNAs	tmRNAs^*c*^	CRISPR
MAFF 211472										
Chromosome	AP024157	3,914,553	66.7	3,673	3	3	3	59	1	1
Megaplasmid	AP024158	2,139,380	66.6	1,726	1	1	1	7	0	0
Total		6,053,933	66.7	5,399	4	4	4	66	1	1
MAFF 211479										
Chromosome	AP024159	3,774,488	66.8	3,514	3	3	3	63	1	0
Megaplasmid	AP024160	2,131,116	66.8	1,738	1	1	1	8	0	0
Total		5,905,604	66.8	5,252	4	4	4	71	1	0
MAFF 311693										
Chromosome	AP024161	3,680,093	67.1	3,441	3	3	3	59	1	0
Megaplasmid	AP024162	2,117,975	66.7	1,741	1	1	1	8	0	0
Plasmid	AP024163	71,852	61.4	89	0	0	0	0	0	0
Total		5,869,920	66.9	5,271	4	4	4	67	1	0

a Detailed classification: MAFF 211472, phylotype I, sequevar 16, race 4, biovar 4; MAFF 211479 and MAFF 311693, phylotype I, sequevar 30, race 4, biovar 4.

b CDS, coding DNA sequences.

c tmRNAs, transfer-messenger RNAs.

### Data availability.

The complete sequences of the R. solanacearum strains have been deposited in the DNA Data Bank of Japan (DDBJ) under BioProject accession number PRJDB9507. The BioSample accession numbers are SAMD00256261, SAMD00256262, and SAMD00256263. The DRA accession numbers are DRX245399/DRX245396, DRX245400/DRX245397, and DRX245401/DRX245398. The GenBank assembly accession numbers are GCA_015698345.1, GCA_015698365.1, and GCA_015698385.1. The genome sequence accession numbers are listed in Table 1. The raw sequencing reads (PacBio and Illumina) were deposited in the DDBJ Sequence Read Archive database under accession number DRA011112.
